# Indocyanine green fluorescence navigation for hepatocellular carcinoma with bile duct tumor thrombus: a case report

**DOI:** 10.1186/s40792-020-01101-7

**Published:** 2021-01-13

**Authors:** Masaru Matsumura, Yasuji Seyama, Hiroyuki Ishida, Satoshi Nemoto, Keigo Tani, Jun Imamura

**Affiliations:** 1 Department of Hepato-Biliary-Pancreatic Surgery, Tokyo Metropolitan Cancer and Infectious Diseases Center Komagome Hospital, Tokyo, Japan; 2 Department of Hepatology, Tokyo Metropolitan Cancer and Infectious Diseases Center Komagome Hospital, Tokyo, Japan

**Keywords:** Hepatocellular carcinoma, Bile duct thrombus, Indocyanine green, Fluorescence navigation

## Abstract

**Background:**

Bile duct tumor thrombus (BDTT) is one of the features of advanced hepatocellular carcinoma (HCC). In the resection of HCC with BDTT, it is important to detect the BDTT tip to decide the appropriate point of bile duct division. In this regard, the efficacy of indocyanine green (ICG) fluorescence navigation has been confirmed for the detection of HCC, whereas its utility for BDTT has not yet been reported. Herein, we describe our experience with right hepatectomy for HCC with BDTT using ICG fluorescence navigation.

**Case presentation:**

A 72-year-old woman had experienced local recurrences of HCC after radiofrequency ablation, with BDTT reaching the confluence of the right anterior branch and posterior branch. Right hepatectomy was planned, and 2.5 mg of ICG was injected one day before surgery. After transection of the liver parenchyma, the right liver was connected with only the right hepatic duct. ICG fluorescence imaging visualized the tip of BDTT in the bile duct with clear contrast; the proximal side (hepatic side) of the right hepatic duct showed stronger fluorescence than the distal side (duodenal side). The bile duct was divided at the distal side of the BDTT border, and the tip of BDTT was recognized into the resected right hepatic duct without laceration. The patient had an uneventful postoperative course and currently lives without recurrences for 6 months.

**Conclusions:**

ICG fluorescence navigation assisted in the precise resection of the bile duct in HCC with BDTT.

## Background

Bile duct tumor thrombus (BDTT) is one of the features of advanced hepatocellular carcinoma (HCC). Although the progression of BDTT is an oncological emergency leading to obstructive jaundice followed by liver failure, complete removal of the tumor may provide long-term survival [1–11]. For the resection of HCC with BDTT localized primarily within the first branch of the bile duct, which is defined as B3 by the liver cancer study of Japan [12], it is important to detect the BDTT tip to decide about the appropriate point of bile duct division. In this regard, the efficacy of indocyanine green (ICG) fluorescence navigation has been confirmed in the detection of HCC, whereas its utility for BDTT has not yet been reported.

Herein, we describe our experience with right hepatectomy for HCC with BDTT using ICG fluorescence navigation.

## Case presentation

A 72-year-old woman was followed up at our institute after radiofrequency ablation (RFA) for HCC. She had a history of hepatitis C infection, which was treated by direct antiviral agent therapy that had provided her with a serological viral response 3 years earlier. Two years earlier, she had developed a solitary HCC with a diameter of 1.8 cm, for which RFA was performed. Follow-up plain computed tomography (CT) revealed a newly developed low-density area around the RFA scar.

**Fig. 1:**
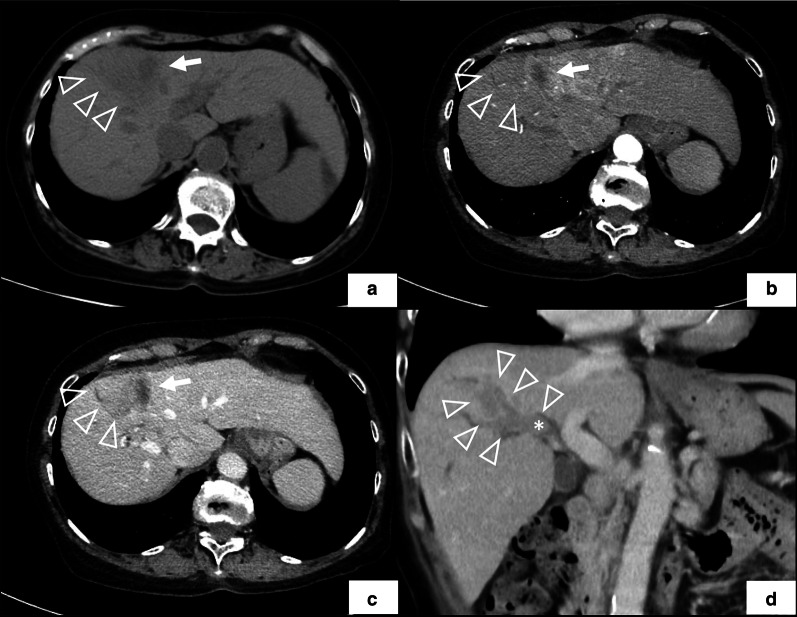
Preoperative dynamic computed tomography study. **a** Low-density area (arrow head) located adjacent to the scar of radiofrequency ablation (arrow) in the plain phase. **b** The image of early phase does not show early staining on the area. **c** The image of late phase visualized a well-defined mass with enhancement on the area. **d** The image of multiplanar reconstruction of the tumor (arrow head) with the bile duct thrombus (asterisk). BDTT continued from the tumor into the right hepatic duct

**Fig. 2:**
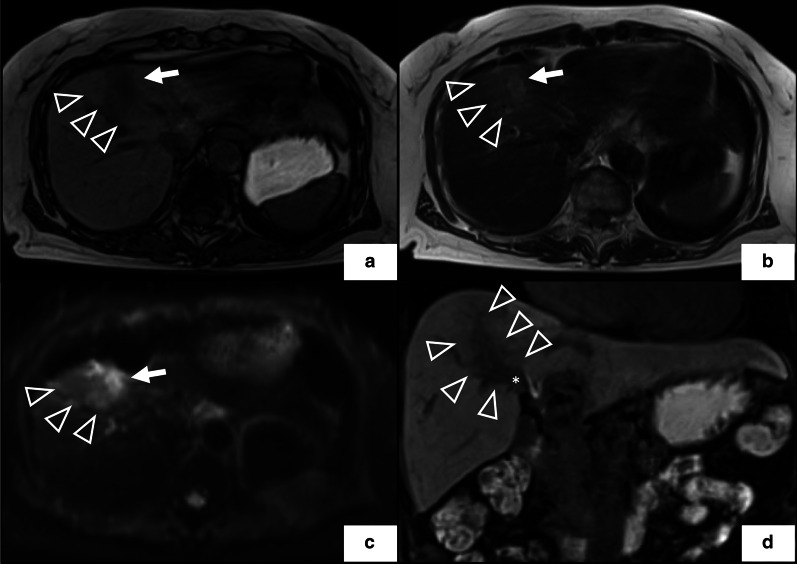
Preoperative magnetic resonance imaging findings. The tumor (arrow head) adjacent to the scar of radiofrequency ablation (arrow) exhibits low intensity on the T1-weighted image (**a**) and a slightly high intensity on T2- (**b**) and diffusion-weighted images (**c**). **d** Hepatobiliary phase of sagittal scan shows a low-intensity mass (asterisk) that continued from the tumor into the right hepatic duct

In the dynamic CT study, the area around the RFA scar in segment 8 did not exhibit early staining in the arterial phase. In the image of the portal phase, a mass was visualized with enhancement in the area, and the area continued into the bile duct of the right anterior section as a well-defined mass. The tip of the mass extended to the confluence of the anterior branch and posterior branch. The tributary of the bile duct in the anterior segment was dilated (Fig. 1). On gadolinium-ethoxybenzyl-diethylene-triaminepentaacetic acid (Gd-EOB) magnetic resonance imaging, the area adjacent to the RFA scar exhibited low intensity on the T1-weighted image and a slightly high intensity on T2- and diffusion-weighted images. The hepatobiliary phase of the sagittal scan revealed a low-intensity mass that continued from the tumor into the right hepatic duct (Fig. 2). Laboratory data disclosed slightly elevated liver enzyme levels, whereas the levels of alpha-fetoprotein and des-gamma-carboxy prothrombin were within normal range. The ICG retention rate at 15 min was 9.6% (Table 1). The Child–Pugh score was 5 points [12].

**Table 1:** Preoperative laboratory data

	Values
White blood cells, /µl	6400
Red blood cells, /µl	4.36 × 10^6^
Platelet, /µl	13.5 × 10^4^
Serum Albumin, g/dl	4.1
Total bilirubin, mg/dl	0.6
Direct Bilirubin, mg/dl	0.2
Aspartate aminotransferase, U/L	66
Alanine aminotransferase, U/L	63
Gamma-glutamyl transferase, U/L	508
Alkaline phosphatase, U/L	785
Urea nitrogen, mg/dl	16
Serum Creatinine, mg/dl	0.65
C-reactive protein, mg/dl	0.15
Prothrombin time, s	9.9
Alpha-fetoprotein, ng/ml	6.0
Des-gamma-carboxy prothrombin, mAU/ml	17.0
Hepatitis C antibody	Positive
ICGR15, %	9.6

*ICGR15* indocyanine green retention rate at 15 min

**Fig. 3:**
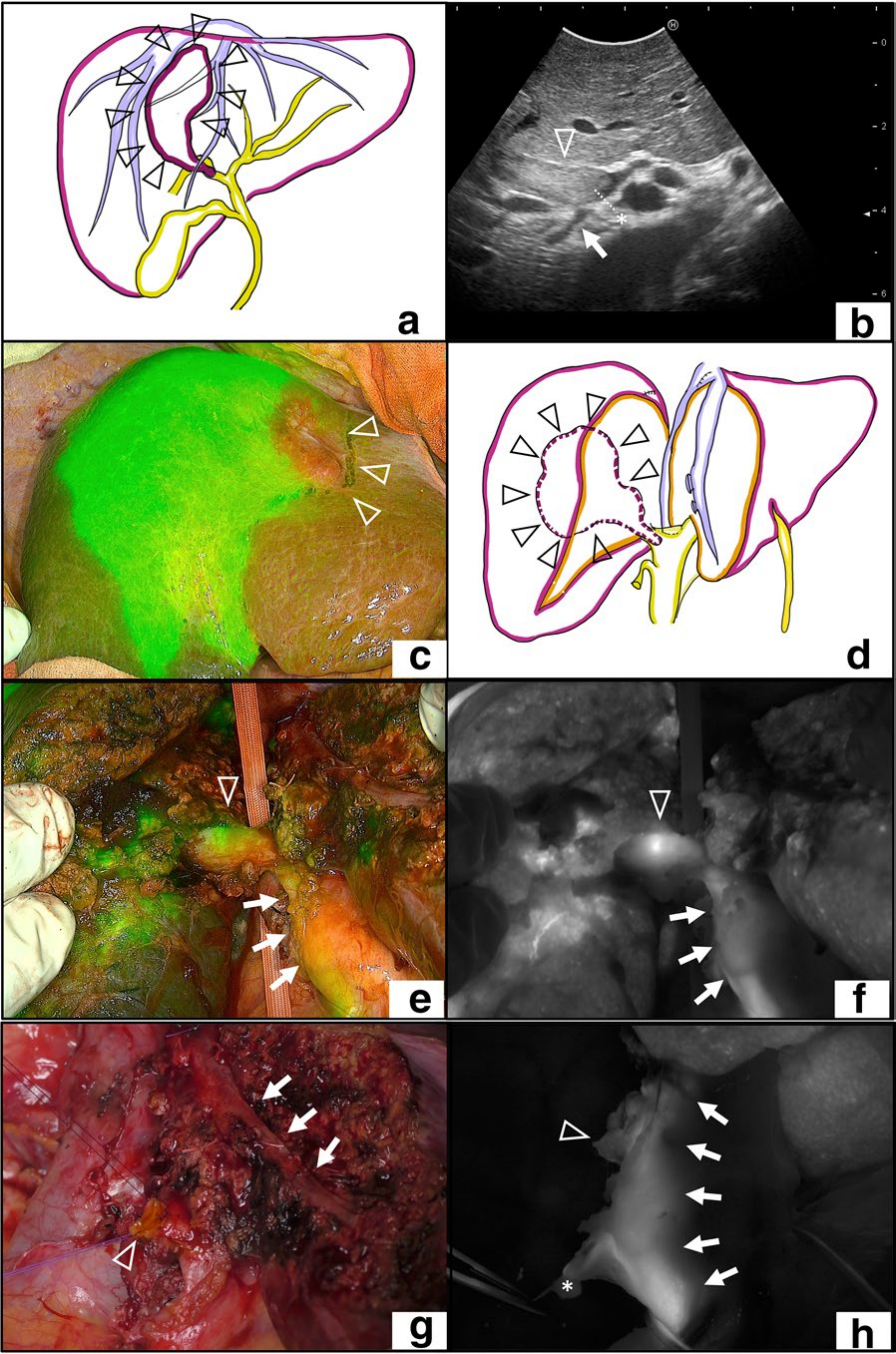
Operative findings. **a** Preoperative scheme of the patient. Triangle: tumor. **b** Indocyanine green (ICG) fluorescence imaging. Right anterior section demonstrated evident fluorescence. Arrow head: serosal deformity from tumor. **c** Intraoperative ultrasonography. The tip of the bile duct tumor thrombus (BDTT) located at the hepatic side of the right hepatic duct. Arrow head: the bile duct of the right anterior branch. Arrow: the posterior branch, Asterisk: the tip of BDTT. **d** Intraoperative scheme after division of liver parenchyma. Triangle: tumor. **e**, **f** ICG fluorescence imaging before the division of the right hepatic duct. The light of emission from the bile duct and BDTT with clear contrast; the proximal side of the right hepatic duct exhibited stronger fluorescence than the proximal side. Arrow head: location of the BDTT tip in the right hepatic duct. Arrow: common hepatic duct. **g** After the removal of surgical specimen. Arrow head: stump of the right hepatic duct. Arrow: middle hepatic vein. **h** ICG fluorescence imaging of the bile duct after resection. Bile duct route from the left hepatic duct to the common hepatic duct is clearly visualized. Arrow head: stump of the right hepatic duct. Arrow: Bile duct route from the left hepatic duct to the common hepatic duct. Asterisk: stump of cystic duct

We made a diagnosis of local recurrences of HCC after RFA, with BDTT reaching at the confluence of the anterior branch and posterior branch, which is defined as B3 by the liver cancer study of Japan [12]. Right hepatectomy was planned for radical resection (Fig. 3a). One day before surgery, 2.5 mg of ICG was injected for intraoperative fluorescence navigation.

**Fig. 4:**
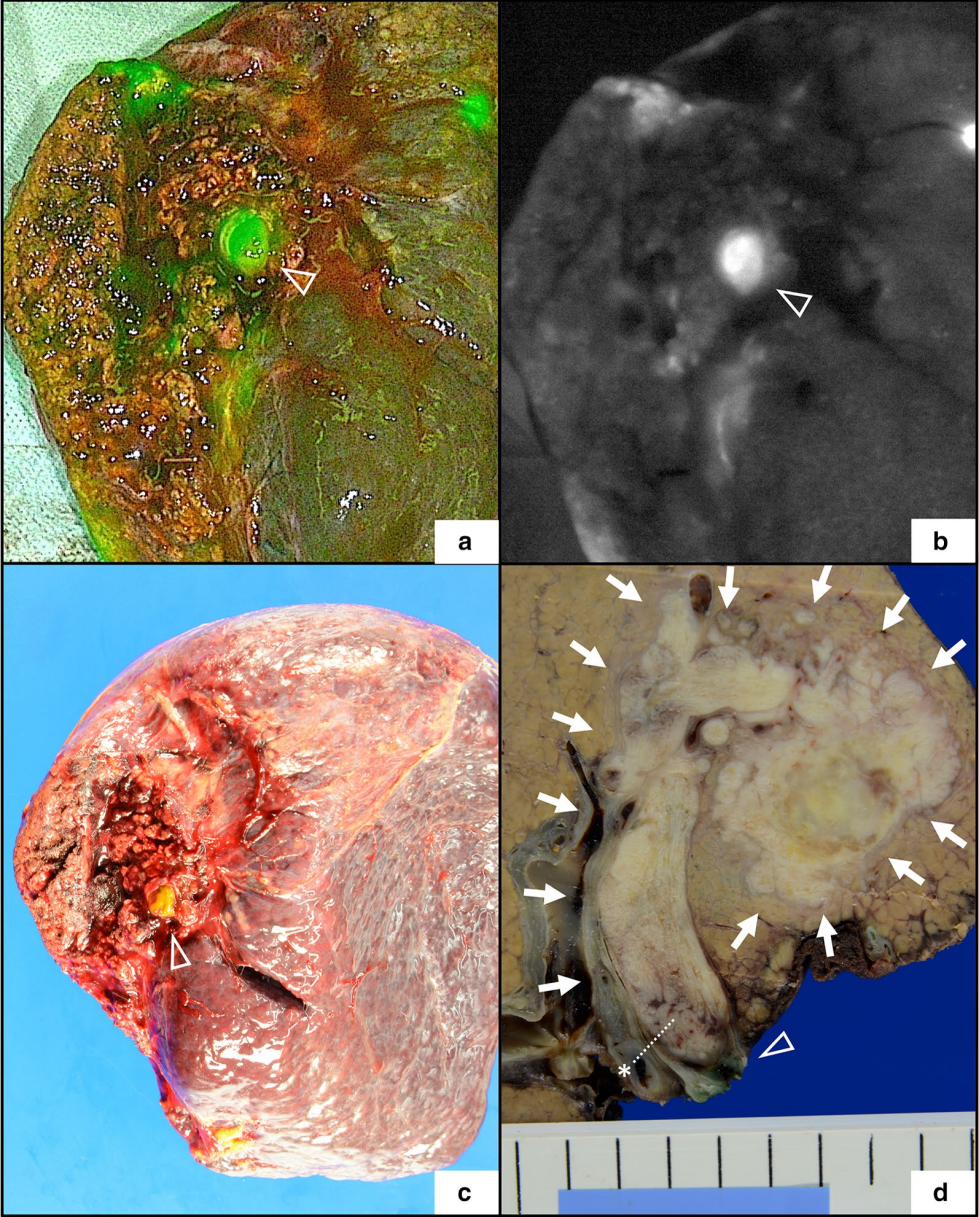
Surgical specimen. **a**, **b** Gross findings of the resected specimen of indocyanine green (ICG) fluorescence imaging (**a** overlay mode, **b** white-black mode). Bile duct tumor thrombus (BDTT) emitted strong fluorescence. Arrow head: The tip of BDTT at the stump of the right hepatic duct. **c** Gross findings of the resected specimen. Arrow head: The tip of BDTT at the stump of the right hepatic duct. **d** Cut surface of the resected specimen with secured surgical margin at the right hepatic duct. Arrow: tumor, asterisk: The tip of BDTT, arrow head: The stump of the right hepatic duct

A J-shaped incision was made. Abdominal exploration did not reveal any unresectable factors. The liver edge was dull, and the parenchyma was slightly hard. The tumor and its RFA scar were recognized from the liver surface as serosal deformity on the right anterior segment. Intraoperative ultrasonography (IOUS) showed that the BDTT tip was located at the hepatic side of the right hepatic duct (Fig. 3b). On observation by the ICG fluorescence imaging system (PINPOINT, Striker, USA), the right anterior section demonstrated evident fluorescence, which reflected impaired excretion of bile due to BDTT (Fig. 3c). After dividing the right hepatic artery and the right branch of the portal vein, transection of liver parenchyma was performed along with a demarcation line on Cantlie’s line. To maintain the surgical margin, the resection line was extended to the left liver near the tumor. Finally, the right liver was connected with the right hepatic duct alone (Fig. 3d). ICG fluorescence imaging demonstrated the light of emission from the bile duct and BDTT with clear contrast; the proximal side (hepatic side) of the right hepatic duct exhibited stronger fluorescence than the distal side (duodenal side), which was compatible with the findings of IOUS before liver transection (Fig. 3e, f). The border was located approximately 8 mm from the confluence of the right and left hepatic duct, which was compatible with the findings of IOUS. Examination by palpation of the BDTT tip on the right hepatic duct was avoided due to its fragility. The bile duct division was made at the distal side of the BDTT border, and the surgical specimen was removed. The tip of BDTT was recognized into the resected right hepatic duct without laceration. The stump of the right hepatic duct was closed by interrupted suture using 4-0 absorbable monofilament threads (Fig. 3g). ICG fluorescence cholangiography performed after resection confirmed the bile duct route from the left hepatic duct to the common hepatic duct (Fig. 3h). The resected specimen demonstrated evident light of emission from BDTT when observed using the ICG fluorescence imaging system (Fig. 4a, b). Gross findings of the resected specimen revealed a confluent multinodular mass with BDTT with a secured surgical margin at the right hepatic duct (Fig. 4c, d). Pathological findings were as follows: St-A, 4 × 3.7 × 2.5 cm, confluent multinodular type, poorly differentiated hepatocellular carcinoma, e.g., fc (−), sf (−), s1, vp1, vv0, va0, b3, and surgical margin was negative for cancer [12].

The patient had an uneventful postoperative course and was discharged on the 13^th^ postoperative day. Currently, she lives without recurrences for 9 months after surgery.

## Discussion

The incidence of BDTT in patients with HCC has been reported to be 1.3–13% [1, 3, 4, 6, 13–15]. Although it is a life-threatening feature of advanced HCC, radical resection for selected candidates provides an impressive prognosis, reaching a 5-year overall survival of 48% as reported in previous studies [1–11]. In the present case, we used ICG fluorescence navigation to identify the location of the BDTT tip at the first-order branch of the bile duct. To our knowledge, this is the first report to describe the usage of ICG fluorescence navigation for the resection of BDTT.

ICG fluorescence observation provided the contrast of the clear border of BDTT after liver transection. As demonstrated in our case, IOUS is effective for the detection of the BDTT tip. However, IOUS performed after liver transection does not provide clear images due to decay of signal by air around the bile duct. Fluorescence navigation could be one of the candidates approaches to overcome this drawback of IOUS. In the present case, the tumor thrombus was visualized within the bile duct based on the discrepancy of the fluorescence intensity between BDTT and bile. This might be based on two characteristics of ICG, i.e., ICG fluorescence cholangiography and ICG retention in HCC tumor cells. ICG cholangiography was first reported in 2009 for laparoscopic cholecystectomy using the characteristics of ICG to excrete into bile. [16] Regarding ICG retention, some HCC has the feature of ICG retention, which aids in tumor detection in intraoperative fluorescence observation [17–19]. In our case, we could resect the right hepatic duct beyond the BDTT. Fluorescence navigation combined with IOUS assisted in the precise complete resection of BDTT as an alternative of direct cholangiography, which facilitated the operative procedure.

Studies discussed on the timing and dosage of ICG administration of intraoperative fluorescence navigation. For detecting liver tumor, the prevailed method is 0.5 mg/kg within several days [17]. A recent study compared several dosages of ICG administration for tumor identification [20]. In the study, the comparison of four different dosage (0.25 mg, 1.25 mg, 2.5 mg, and 3.75 mg) one day prior to surgery and original method was conducted, and 2.5 mg one day prior to surgery had balanced results; the identification rate: 78.3%, false positive rate: 9.1%. For fluorescence cholangiography, original method was 2.5 mg before surgery [16]. In this regard, studies discuss that earlier timing may offer clear contrast between bile duct and background [21, 22]. To weaken the fluorescence of bile for the contrast between BDTT and bile, we consider small amount of ICG (2.5 mg) one day before surgery is reasonable. In the current case, our protocol provided clear border of BDTT in the bile duct.

The procedure of resection is one of the factors that influences recurrences in HCC with BDTT. Several studies have reported that thrombectomy was associated with local recurrences [5, 8, 11]. Although the results were not conclusive due to their retrospective feature, these results imply that thrombectomy might not be recommended for HCC with BDTT in case the patient’s condition permitted extensive surgery. On the other hand, BDR is associated with high risks for future complications in locoregional treatment in the remnant liver [23, 24]. From this perspective, we attempted to design the cut line of the bile duct beyond the tip of BDTT.

It is conceivable that fluorescence navigation might be indicated for other patients with BDTT that exhibits different features. One issue is that the detection of BDTT tip by fluorescence navigation might be beneficial even in B4 disease. It supposes that ICG fluorescence navigation helps decide the division line of the bile duct in both bile duct resection and thrombectomy. Another issue is that the BDTT tip might be detectable irrespective of whether the tumor exhibits fluorescence. A liver tumor does not always exhibit fluorescence by itself. Ishizawa et al. reported that 30 of 63 HCCs were only of partial fluorescence type or rim fluorescence type, and all the metastatic nodules were of rim fluorescence type [17]. In our case, the BDTT exhibited strong fluorescence. However, even in patients with BDTT without ICG fluorescence, the BDTT tip might be detectable as a defect in the bile duct based on the discrepancy of fluorescence. Actually, retention of ICG in BDTT was not histologically proved in our case. Further study is required to evaluate the efficacy of ICG fluorescence navigation for other features of a tumor with BDTT.

## Conclusions

We have described a case of radical resection for HCC with BDTT. ICG fluorescence navigation assisted in the precise resection of the bile duct in HCC with BDTT.

## Data Availability

Not applicable.

## Abbreviations

BDTT: Bile duct tumor thrombus
HCC: Hepatocellular carcinoma
RFA: Radiofrequency ablation
ICGR15: Indocyanine green retention rate at 15 min
IOUS: Intraoperative ultrasonography
